# Clinical Study of Cutaneous Manifestations in Neonates in A Tertiary Care Center in Eastern Nepal: A Descriptive Cross-sectional Study

**DOI:** 10.31729/jnma.8783

**Published:** 2024-10-31

**Authors:** Anil Timilsina, Nisha Keshary Bhatta, Nidhi Shah

**Affiliations:** 1 Department of Dermatology Madan Bhandari Hospital and Trauma Center, Morang, Nepal; 2 Department of Pediatrics and Division of Neonatology, B.P. Koirala Institute of Health Sciences, Dharan, Nepal; 3 Department of Dermatology and Venereology, B.P. Koirala Institute of Health Sciences, Dharan, Nepal

**Keywords:** *maternal age*, *newborn*, *physiological*, *skin manifestations*

## Abstract

**Introduction:**

In the neonatal period, a variety of cutaneous symptoms are frequent. To minimize unneeded therapy and to reduce parents' stress, it's important to distinguish between transient and pathological newborn dermatoses. The purpose of this study is to describe the clinical pattern of cutaneous lesions in neonates.

**Methods:**

In this cross-sectional descriptive study, we enrolled 512 neonates born and admitted in maternal child health care ward and Post Natal ward of a tertiary health care center irrespective of cutaneous manifestations. Convenience sampling technique was used. They were examined till their stay in hospital and followed up weekly via telephone for development of dermatological manifestations.

**Results:**

Out of 512 neonates examined, 415 (81.05%) of neonate had at least one cutaneous manifestation, 393 (94.69%) had physiological skin lesions, 22 (5.31%) had pathological skin lesions. The most common physiological skin manifestations were sebaceous gland hyperplasia, mongolian spot, ETN, milia and epstein pearl. The pathological cutaneous manifestations though least common comprised of cradle cap, napkin dermatitis and bacterial infections. Most of the lesions were present within 24 hours of birth and many of them were physiological transient lesion.

**Conclusions:**

In conclusion, our study highlights the high prevalence of cutaneous manifestations in neonates, with the majority being physiological in nature. Medical personnel must be well versed in the skin changes of neonates which will further help prevent unnecessary treatment and identify life threatening situations that require additional attention.

## INTRODUCTION

Cutaneous manifestations in newborns can be broadly classified into physiological, transient eruptions, birth marks, inherited disorders, cutaneous infections and pathological cutaneous manifestations.^1^ The majority of neonatal cutaneous conditions are transient physiological, reversible and spontaneously regressing lesions that require no therapy and unnecessary investigations; nevertheless, concerned parents frequently need reassurance of the benign nature of these lesions.^2,3^

Furthermore, there are few studies that have highlighted the prevalence and proportion of neonate cutaneous manifestation with different maternal and neonatal factors.^4,5^ So the aim of this study is to study the cutaneous manifestations in neonates.

## METHODS

This cross-sectional descriptive study was conducted in B.P. Koirala Institute of Health Sciences, Dharan, Nepal. The study duration was of 6 month from March 1, 2021 to August 30, 2021. Cases were enrolled from Maternal child health care ward and post-natal ward of Department of Obstetrics and Nursery and Neonatal intensive care unit(NICU) of Department of Pediatrics. Ethical approval was taken from the Institutional Review Committee (IRC/1885/020).

The proportion of cutaneous manifestation among newborns was recorded from 40%-75%^6-8^ based on multiple prospective studies done before. And taking the 75% of sample size calculated from given formula.

The formula used was as follows:


$$\begin{array}{ll} \text{n} & ={\text{Z}}^{2}\times \frac{\text{p}\times \text{q}}{{\text{e}}^{\text{2}}}\\ & =1.96^{2}\times \frac{\text{0.75}\times \text{ (1}-\text{0.75)}}{0.0375^{2}}\\ & =512 \end{array}$$

Where:

n is the minimum required sample size for an infinite population.
z represents the z-value (1.96) at a 95% Confidence Interval (CI).
p is the estimated prevalence of DH in the general population (75).^6^
q = 1-p
e is the margin of error taken as 5% of estimated prevalence 75% as 3.75).

Hence the sample size needed to be enrolled is 512. Convenience sampling technique was used.

Examination of newborns at Post Natal and Maternal and Child Health Care ward of Department of Obstetrics and Nursery and NICU of Department of Pediatrics were done by Dermatologist twice weekly (i.e., Sunday and Wednesday) and newborns on those specific days were enrolled in the study irrespective of presence of dermatological manifestations. They were examined till their stay in hospital and followed up weekly via telephone for development of dermatological manifestations. In addition, the parents were advised to bring the neonate in OPD of Department of Dermatology and Venereology if any skin manifestations appeared within 4 weeks of age.

Newborns in Post Natal and Maternal and child health care ward of Department of Obstetrics, neonates admitted in Nursery, Neonatal ward, and Neonatal intensive care unit of pediatrics and neonates visiting OPD in Department of Dermatology and Venereology were included in the study. Patients whose parents/guardians were not willing to consent to participate and presence of any illness keeping the neonate immobilized at the Neonatal Intensive Care Unit (NICU), thus preventing a daily appropriate dermatological examination were excluded from the study. A detailed history of mother and child were taken as well as clinical examination and relevant investigations were done. The data were entered in a proforma and later analyzed.

A written consent was taken from all parents. All obtained data were entered in Microsoft excel and analyzed by SPSS 16. Descriptive data was tabulated in frequency and percentage.

## RESULTS

A total of 512 neonates were enrolled in the study from March, 2021 to August, 2021 out of which 415 (81.05%) had at least one cutaneous manifestation. Out of 415 neonates, the newborn with only one number of cutaneous lesion were 256 (61.68%), while those with two number and three number of cutaneous lesion number were 151 (36.38%) and 8 (1.92%) respectively. 393 (94.69%) had physiological skin lesions, 22 (5.30%) had pathological skin lesions. Out of 415 neonates with cutaneous manifestations, the most common physiological manifestations were sebaceous gland hyperplasia was 169 (40.90%) mongolian spot 119 (28.60%), Erythema toxicum neonatarum 63 (15.17%), milia 70 (16.87%) and epstein pearl 43 (10.36%) were the most common physiological manifestations whereas bacterial infections, cradle cap 4 (0.96%) and napkin dermatitis 4 (0.96%) were pathological cutaneous manifestations (Tables 1 and 2).

**Table 1:** Demographic profile of neonates (n=512)

Characteristics	n (%)
**Gender**	
Male	273 (53.52)
Female	239 (46.68)
**Religion**	
Hindu	475 (92.77)
Buddhist	16 (3.13)
Muslim	14 (2.73)
Christian	7 (1.37)
**Birth weight**	
<2.5 kg	140 (27.34)
2.5-3.5 kg	319 (62.31)
>3.5 kg	53 (10.35)

Four neonatal and four maternal factors were studied in our study (Table 3). Out of 415 neonates with cutaneous manifestations 304 (73.25%) had cutaneous manifestations within 24 hours of delivery, while 86 (20.72%) had cutaneous manifestations between 24 hours to 72 hours. Similarly, 15 (3.61%) of neonates had cutaneous manifestations in the period below 72 hours to 1^st^ week, 5 (1.20%) in the 2^nd^ week, 3 (0.72%) in the 3^rd^ week and 2 (0.48%) in the 4^th^ week (Table 4).

**Table 2:** Frequency and Pattern of cutaneous manifestations in neonate (n=425)

Cutaneous manifestations	Percentage n (%)
**Physiological skin lesion**	
Transient non infective cutaneous manifestations	N (%)
Sebaceous gland hyperplasia	169 (40.72)
Mongolian spot	119 (28.67)
Milia	70 (16.87)
Erythema toxicum neonatorum	63 (15.18)
Epstein pearls	43 (10.36)
Hypertrichosis	17 (4.1)
Genital pigmentation	14 (3.37)
Physiological scaling	13 (3.13)
Miliaria rubra	5(1.2)
Physiological Jaundice	4 (0.96)
Miliaria crystillina	4 (0.96)
Transient neonatal pustular melanosis	2 (0.48)
Suckling blister	1 (0.24)
**Birth mark**	
Salmon patch	29 (6.99)
Cafe au lait macule	4 (0.96)
Congenital melanocytic nevus	2 (0.48)
Linear and whorled nevoid hyper melanosis	1 (0.24)
**Pathological cutaneous lesion**	
**Birth trauma**	
Erosion	3 (0.72)
Bruise	2 (0.48)
Laceration	1 (0.24)
**Eczematous eruptions**	
Cradle cap	4 (0.96)
Napkin dermatitis	4 (0.96)
Developmental defects	
Cleft lip	1 (0.24)
Upper eyelid coloboma	1 (0.24)
Accessory tag	1 (0.24)
**Bacterial infections**	
Omphalitis	4 (0.96)
Ecthyma gangrenosum	1 (0.24)

Among the maternal factors, 127 (30.60) of the 415 neonates with cutaneous manifestations had a mother who had disease during pregnancy, while 288 (69.40) had a mother who had no disease during pregnancy (Tables 4 and 5).

Physiological scaling was found to be prevalent in females, while genital hyperpigmentation was found to be prevalent in male. Mongolian spot was found often in term neonates in the study. Physiological scaling was also demonstrated in many post-term neonates. In term neonates, however, sebaceous gland hyperplasia, milia, epstein pearl, ETN, salmon patch, genital hyperpigmentation, and hypertrichosis were all common. Mongolian spot and hypertrichosis were detected frequently in neonates delivered vaginally, whereas epstein pearls and ETN 41(65.07) were frequently found in neonates born from LSCS.

**Table 3:** Number of cases with Neonatal and Maternal factors (n=415)

Variables	n (%)
**Neonatal factors**	
**Age of onset of lesion**	
Birth-24 hours	304 (73.25)
>24 hours- 72 hours	86 (20.72)
>72hours- 1^st^ week	15 (3.61)
1^st^ week- 2^nd^ week	5 (1.20)
2^nd^ week- 3^rd^ week	3 (0.72)
3^rd^ week- 4^th^ week	2 (0.48)
**Gender**	
Male	221 (53.25)
Female	194 (46.75)
**Birth weight**	
< 2.5 kg	113 (27.23)
2.5-3.5 kg	259 (62.41)
> 3.5 kg	43 (10.36)
**Maturity**	
Preterm	39 (9.39)
Term	338 (81.45)
Post term	38 (9.16)
**Maternal factors**	
**Maternal age**	
< 20 years	35 (8.43)
20-30 years	311 (74.94)
>30 years	69 (16.62)
**Mode of delivery**	
Vaginal	228 (54.94)
Caesarean	187 (45.06)
**Number of pregnancies**	
Multigravida	211 (50.84)
Primigravida	204 (49.16)
**Maternal disease during pregnancy**	
Gestational DM	41 (9.88)
Gestational HTN	15 (3.61)
Pre-eclampsia	18 (4.33)
Eclampsia	13 (3.13)
Anemia	24 (5.78)
Thyroid disorder	10 (2.41)
Others	6 (1.44)

Gestational diabetes mellitus, eclampsia, pre-eclampsia and anemia were present in the mother of neonate who had miliaria rubra. The other cutaneous manifestation was also associated with presence of maternal disease during pregnancy.

**Table 4:** Distribution of cases according to age of onset of lesion

S. N	Cutaneous lesion	Birth-24hrs n (%)	>24 hours-72 hours n (%)	>72 hours-1st week n (%)	1st week-2nd week n (%)	2nd week-3rd week n (%)	3rd week-4th week n (%)
1.	Sebaceous gland hyperplasia (n=169)	163 (96.45)	6 (3.55)	-	-	-	-
2.	Milia (n=70)	62 (88.57)	8 (11.43)	-	-	-	-
3.	Mongolian spot (n=119)	111 (93.27)	8 (6.73)	-	-	-	-
4.	Epstein pearls (n=43)	42 (97.67)	1 (2.33)	-	-	-	-
5.	ETN (n=63)	4 (6.35)	52 (82.54)	7 (11.11)	-	-	-
6.	Salmon patch (n=29)	21 (72.41)	5 (17.24)	1 (3.44)	1 (3.44)	1 (3.44)	-
7.	Physiological scaling (n=13)	10 (76.92)	3 (23.08)	-	-	-	-
8.	Hypertrichosis (n=17)	15 (88.23)	2 (11.77)	-	-	-	-
9.	Genital Hyperpigmentation (n=14)	12 (85.71)	2 (14.29)	-	-	-	-
10.	Cafe au lait macule (n=4)	3 (75)	1 (25)	-	-	-	-
11.	Physiological Jaundice (n=4)	-	4 (100)	-	-	-	-
12.	Miliaria rubra (n=5)	-	-	2 (40)	1 (20)	1 (20)	1 (20)
13.	Miliaria crystallina (n=4)	1 (2)	1 (25)	1 (25)	-	1 (25)	-
14.	Cradle cap (n=4)	-	-	-	2 (50)	1 (25)	1 (25)
15.	Birth Trauma (n=6)						
	i. Bruise	2 (33.33)	-	-	-	-	-
	ii. Erosion	3 (50)	-	-	-	-	-
	iii. Laceration	1 (16.67)	-	-	-	-	-
16.	Congenital melanocytic nevus (n=2)	2 (100)	-	-	-	-	-
17.	Transient neonatal pustular melanosis (n=2)	-	2 (100)	-	-	-	-
18.	Napkin dermatitis (n=4)	-	-	2(50)	-	-	2(50)
19.	Omphalitis (n=4)	-	-	2 (50)	1 (25)	1 (25)	-
20.	Upper eyelid coloboma (n=1)	1 (100)	-	-	-	-	-
21.	Accessory tag (n=1)	1 (100)	-	-	-	-	-
22.	Ecthyma gangrenosum (n=1)	-	-	1 (100)	-	-	-
23.	Linear and whorled nevoid hyper melanosis (n=1)	1 (100)	-	-	-	-	-
24.	Suckling blister (n=1)	1 (100)	-	-	-	-	-
25.	Cleft lip (n=1)	1 (100)	-	-	-	-	-

**Table 5:** Distribution of cutaneous manifestations according to each maternal disease during pregnancy

S. N	Cutaneous lesion	Gestational HTN	Gestational DM	Preeclampsia	Eclampsia	Anemia	Thyroid disorder	Others
1.	Sebaceous gland hyperplasia (n=169)	5 (13.89)	11 (30.55)	4 (11.11)	4 (11.11)	8 (22.22)	3 (8.33)	1 (2.77)
2.	Milia (n=70)	3 (17.64)	4 (23.53)	3 (17.64)	1 (5.89)	3 (17.64)	2 (11.76)	1 (5.89)
3.	Mongolian spot (n=119)	1 (5.26)	5 (26.31)	4 (21.05)	2 (10.52)	4 (21.05)	2 (10.52)	1 (5.26)
4.	Epstein pearls (n=43)	1 (10)	4 (40)	1 (10)	1 (10)	1 (10)	1 (10)	1 (10)
5.	ETN (n=63)	1 (11.11)	3 (33.33)	1 (11.11)	1 (11.11)	1 (11.11)	1 (11.11)	1 (11.11)
6.	Salmon patch (n=29)	1 (11.11)	3 (33.33)	1 (11.11)	1 (11.11)	1 (11.11)	1 (11.11)	1 (11.11)
7.	Physiological scaling (n=13)	-	1 (25)	1 (25)	1 (25)	-	1 (25)	-
8.	Hypertrichosis (n=17)	-	2 (50)	-	1 (25)	1 (25)	-	-
9.	Genital Hyperpigmentation (n=14)	1 (25)	2 (50)	1 (25)				
10.	Cafe au lait macule (n=4)	-	2 (66.66)	1 (33.34)	-	-	-	-
11.	Physiological Jaundice (n=4)	-	2 (40)	1 (20)	1 (20)	1 (20)	-	-
12.	Miliaria rubra (n=5)	-	-	-	-	4 (100)	-	-
13.	Miliaria crystallina (n=4)	-	2 (50)	-	-	2 (50)	-	-
14.	Birth Trauma (n=6)							
	i. Bruise	2 (33.33)	-	-	-	-	-	-
	ii. Erosion	3 (50)	-	-	-	-	-	-
	iii. Laceration	1 (16.67)	-	-	-	-	-	-
15.	Napkin dermatitis (n=4)	1 (100)	-	-	-	-	-	-
16.	Omphalitis (n=4)	-	-	-	-	1 (100)	-	-
17.	Upper eyelid coloboma (n=1)	-	1 (100)	-	-	-	-	-

The presence of milia was commonly seen in neonates whose mothers had gestational hypertension. Physiological jaundice, on the other hand, was found among neonates whose mothers had gestational diabetes mellitus. Miliaria crystallina 4 (100%) and cradle cap 2 (50%) were more common in neonates whose mothers had anemia.

## DISCUSSION

In our study, 415 neonates had cutaneous lesion with frequency of 81.05%. In the study done in Nepal the prevalence of neonatal dermatoses had been reported from 63% to 93.8% comparable to the finding of our study.^9,10^ Prevalence of neonatal cutaneous findings in literature has been reported to be from 40% to 99.3%.^1,9-12^ The most common cutaneous manifestations were physiological skin lesions which were seen in 94.69% neonates and pathological skin lesions were seen in 5.30% neonates in our study. The physiological skin lesion and pathological skin lesions were 84.4% and 12.6% respectively found in a study done by Agrawal G, 2012.^11^ The frequency of pathological skin lesions was higher in this study most probably because the babies were examined over a period of 6 months. However, in our study we have examined the newborn till 28 days of life.

SGH, MS, salmon patch, epstein pearl, hypertrichosis, genital hyperpigmentation, and birth trauma were the most manifestations seen within 24 hours of birth, accounting for 73.25 % of cases. ETN was the most common cutaneous manifestations seen in the period below 24 hours to 72 hours, but other manifestations such as physiological jaundice and transient neonatal pustular melanosis were also common. Miliaria rubra and miliaria crystallina were commonly presented in period below 72 hours to 1 week whereas omphalitis, cradle cap, and napkin dermatitis were among the cutaneous manifestations seen in the second, third and fourth weeks which accounted for 0.80%, 0.80% and 0.40% of cases respectively. This suggests that the majority of physiological cutaneous manifestations occurred within the first 24 hours.

The most prevalent physiological cutaneous manifestations found in our study were sebaceous gland hyperplasia (40.90). The study conducted by Shrestha A, 2018 and Basnet S, 2016 did not find sebaceous gland hyperplasia in their study. However, studies in other part of the world found that sebaceous gland hyperplasia affects 31.8% to 66.6% of people.^12,13,14^ The frequency of sebaceous gland hyperplasia in our study was 40.9%, which was similar to study conducted by Moosavi Z, 2006.^7^ Our study found that Sebaceous gland hyperplasia appeared mostly within 24 hours of delivery. Other studies have not established any association between cutaneous manifestations and the onset of the lesion. Haveri FTTS, 20145 and Moosavi Z, 20067 found that sebaceous gland hyperplasia was more common in term neonates, which was similar to our study.

The mongolian spot was the second most prevalent cutaneous manifestation in our study, accounting for 28.6% of all cases. The presence of mongolian spot in neonates varied from 10%-89% in different studies.^1,3,7,9,10,12,15,16^ The study done by Basnet S, 2016^10^ found a mongolian spot in 29% of neonate which was similar to our study. However, another study done in Nepal by Shrestha A, 2018^9^ found a mongolian spot in 64%. The discrepancy could be related to the fact that Nepal has only a few studies and could be another reason of sampling variation. In our study, the age of onset of mongolian spot was within 24 hours after delivery in 93.2%. Few other studies had explored that the mongolian spot was usually seen from birth.^16^ In a study by Kulkarni ML, 1996^17^ and Sachdeva et al, 2002^18^ the mongolian spot was shown to be more prominent in term neonates and neonates weighing more than 2.5 kg, which was similar to our findings. Ferahbas A et al, 2009^12^ found the mongolian spot to be more common in older and larger babies. Kruger EMM et al,2019^13^ also found the mongolian spot to be more commonly in neonate with vaginal delivery but it was not statistically significant.

Milia were the third most prevalent cutaneous manifestations in our study, occurring 16.7% of the cases. The study conducted by Haveri FTTS, 2014^5^ in India found the frequency of milia to be 18.3% which was similar to our study.

The frequency of ETN in our study was 15.1%. The incidence of this particular lesion ranged from 1.3 to 46.8% in other studies.^1,5,19,10,9^ In our study, the onset of ETN was frequently seen after 24 hours. According to Hogade S, 2016^2^ the majority of babies develop a lesion during the 2^nd^ and 3^rd^ day of life, which is similar to our findings. Study conducted by Ekiz O et al, 2013^20^ stated that the ETN was more commonly seen in neonates delivered by LSCS similar to our study.

In our study, the prevalence of epstein pearl was 10.3%. According to other studies, epstein pearl was the most frequent skin lesion, with an incidence ranging from 47.8% to 83%.^1,11,18,21^ Because the lesion was more common in Caucasians and Whites, it may explain why there are fewer cases in our studies. Epstein pearl was found to appear within 24 hours of birth, which was similar in our study. The epstein pearl statistically correlated with birth weight, according to Ahsan U et al, 2010^1^ It was more common in babies who were born with a higher birth weight. Similarly, our study also found the greater frequency of epstein pearl in larger birth group.

The frequency of salmon patch found in our study was 7% of all cutaneous manifestations. In our study, we found that salmon patch appeared more commonly within 24 hours and it was prevalent in female term neonates. Reginatto FP et al, 2017^22^, on the other hand, discovered a positive link between salmon patch and gestational age in their study.

The lanugo hair or hypertrichosis found in our study was 4.0%. Virupakshappa T et al, 2019^23^ found 5% of lanugo hair in their study which was comparable to our study.

Genital hyperpigmentation was present in 3.5% cases. Virupakshappa T et al, 2019^23^ found scrotal hyperpigmentation in 11.8% in their study whereas Kruger EMM et al, 2019^13^ found genital hyperpigmentation in 11.1%. The discrepancy in results could be attributed to the ethnic and geographic variations. We found genital hyperpigmentation more common in male newborns in our study. Virupakshappa T et al, 2019^23^ also discovered that male newborns had increased genital hyperpigmentation.

Physiological scaling was another manifestation found in our study with 3.3% frequency. According to study conducted in various nations, the prevalence of physiological scaling or desquamation ranged from 7.6% to 18%.^2,3^ In our study, physiological scaling appeared within 24 hours of birth and was most common in post-term newborns. Haveri FTTS, 2014^5^, and Sadana DJ et al, 2014^24^ found that physiological scaling was most common in term and post term newborns, which was similar to our findings.

In our study, miliaria rubra and miliaria crystallina were seen in 1.36 % and 1.16 % cases respectively. Milaria prevalence varies amongst literatures, ranging from 1.7% to 28.3%.^9,10,18^

Another cutaneous manifestation found in our study was physiological jaundice, which had a frequency of 1.16%. Icterus was recorded in 3.33 % of the newborns in another study by Jain N et al, 2014^25^ which was similar to ours.

Another cutaneous manifestation observed in our study was cafe au lait macule, which accounted for 1.16 % of all cutaneous manifestations. Haveri FTTS, 2014^5^ found the prevalence of 1.3% of cafe au lait macule in their study which was similar to our study.

The other birthmarks found in our study were congenital melanocytic nevus which were 2 (0.4) in number. Birth trauma, which was 1.16 % in our sample, was another noteworthy cutaneous manifestation. It manifested as bruising, laceration, and erosion. Among them, erosion was the most common.

We also found transient neonatal pustular melanosis in our study which was 0.5%. Ferahbas A et al, 2009^12^ also found similar frequency of transient neonatal pustular melanosis i.e. 0.3%.

Cradle cap and napkin dermatitis were the most common eczematous conditions in our study with 0.8% and 0.4% cases respectively. In our study, 0.8 % of the participants had omphalitis. According to Ahsan U et al, 2010^1^ and Virupakshappa T et al, 2019^23^, omphalitis occurs in 8.0 % and 3.2 % of people, respectively. The low frequency in our study could be attributed to good umbilicus care.

Upper eyelid coloboma, accessory tag, cleft lip, ecthyma gangrenosum, linear and whorled nevoid hypermelanosis, and suckling blister were detected in one case each in our study.

The prevalence of maternal illness in our study was 30.6%. The prevalence was similar to the study conducted by Boccardi D et al, 2007^4^ i.e. 32.3%. In our study, we found that gestational HTN was common in neonates with milia. However, Reginatto FP et al, 2017^22^ discovered that skin erythema and genital hypertrophy were common in mothers of newborns with gestational HTN, and that this was statistically significant. We also found in our study that physiological jaundice was commonly seen in mothers of newborns with gestational diabetes mellitus.

Miliaria crystallina and cradle cap were identified more frequently in newborns whose mothers had anemia during pregnancy in our study. The cause behind this, however, is unknown.

Since it is a single center study, the results do not represent the situation in general population due to possible selection bias. Due to the COVID pandemic, many patients were contacted via telemedicine, which may have hampered clinical assessment which are limitations of this study.

## CONCLUSIONS

This study highlights the high prevalence of cutaneous manifestations in neonates, with the majority being physiological and transient in nature. The findings emphasize the importance of recognizing common physiological skin conditions such as sebaceous gland hyperplasia, Mongolian spots, erythema toxicum neonatorum, milia, and Epstein pearls to avoid unnecessary treatments and alleviate parental concerns. Although pathological skin lesions were relatively rare, their identification and differentiation from physiological lesions remain crucial for appropriate management. Early detection and proper education about these skin conditions can significantly contribute to better neonatal care and reduced parental anxiety.
